# Genomic identification, characterization and differential expression analysis of *SBP-box* gene family in *Brassica napus*

**DOI:** 10.1186/s12870-016-0852-y

**Published:** 2016-09-08

**Authors:** Hongtao Cheng, Mengyu Hao, Wenxiang Wang, Desheng Mei, Chaobo Tong, Hui Wang, Jia Liu, Li Fu, Qiong Hu

**Affiliations:** Oil Crops Research Institute of Chinese Academy of Agricultural Sciences, Key Laboratory of Biology and Genetic Improvement of Oil Crops, Ministry of Agriculture,, No.2 Xudong 2nd Road, Wuhan, 430062 People’s Republic of China

**Keywords:** SBP-box, SQUAMOSA promoter binding protein, Transcription factor, *Brassica napus*

## Abstract

**Background:**

*SBP-box* genes belong to one of the largest families of transcription factors. Though members of this family have been characterized to be important regulators of diverse biological processes, information of *SBP-box* genes in the third most important oilseed crop *Brassica napus* is largely undefined.

**Results:**

In the present study, by whole genome bioinformatics analysis and transcriptional profiling, 58 putative members of *SBP-box* gene family in oilseed rape (*Brassica napus* L.) were identified and their expression pattern in different tissues as well as possible interaction with miRNAs were analyzed. In addition, *B. napus* lines with contrasting branch angle were used for investigating the involvement of *SBP-box* genes in plant architecture regulation. Detailed gene information, including genomic organization, structural feature, conserved domain and phylogenetic relationship of the genes were systematically characterized. By phylogenetic analysis, BnaSBP proteins were classified into eight distinct groups representing the clear orthologous relationships to their family members in Arabidopsis and rice. Expression analysis in twelve tissues including vegetative and reproductive organs showed different expression patterns among the *SBP-box* genes and a number of the genes exhibit tissue specific expression, indicating their diverse functions involved in the developmental process. Forty-four *SBP-box* genes were ascertained to contain the putative miR156 binding site, with 30 and 14 of the genes targeted by miR156 at the coding and 3′UTR region, respectively. Relative expression level of miR156 is varied across tissues. Different expression pattern of some *BnaSBP* genes and the negative correlation of transcription levels between miR156 and its target *BnaSBP* gene were observed in lines with different branch angle.

**Conclusions:**

Taken together, this study represents the first systematic analysis of the *SBP-box* gene family in *Brassica napus*. The data presented here provides base foundation for understanding the crucial roles of *BnaSBP* genes in plant development and other biological processes.

**Electronic supplementary material:**

The online version of this article (doi:10.1186/s12870-016-0852-y) contains supplementary material, which is available to authorized users.

## Background

Transcription factors play a critical role in the life-cycle of plants by activating or suppressing the expression of different target genes [1]. The SQUAMOSA promoter-binding protein (SBP) box family represents one of the transcription factor families characterized by a highly conserved SBP domain, 76 amino acids in length [2–4]. Since the first *SBP-box* gene was identified in *Antirrhinum majus*, many such genes have been characterized from different plant species, thus identifying a moderately sized gene family. Sixteen *SBP-box* genes have been identified in model plant Arabidopsis and many genes have also been characterized in worldwide agriculturally important crops such as rice (*Oryza sativa*) and maize (*Zea mays*) [5–7]. The *SBP-box* genes have been shown to influence many aspects of development including leaf and trichome development, vegetative and reproductive phase transition, plant hormone signaling transduction and other physiological processes [8–15].

Among the identified *SBP-box* genes, many were proven to play essential roles in diverse development processes. Transgenic plants that constitutively express Arabidopsis gene *SPL3* exhibited very early flowering and frequent morphology changes [16]. Arabidopsis *spl8* mutants show altered pollen sac development and overexpression of *SPL8* influences plant fertility by mediating GA dependent signaling pathway [9, 17]. In addition, *SPL8* and other *SPL* genes control gynoecium patterning through interference with auxin homeostasis [18]. *AtSBP7* is a central regulator for copper homeostasis in Arabidopsis [19]. *AtSPL2*, *AtSPL10* and *AtSPL11* in Arabidopsis have been demonstrated to control morphological changes associated with shoot maturation in the reproductive phase [20]. *BraSPL9-2* is the target of microRNA bra-miR156 and controls the heading time of Chinese cabbage [21]. Besides the important roles reported in dicot plants, *SBP-box* genes in monocot plant, such as rice and maize, were also shown to modulate essential developmental processes. Higher expression of *OsSPL14* in the reproductive stage promotes panicle branching and higher grain yield in rice, suggesting the important roles of *SPL* genes in plant architecture regulation [22, 23]. Maize transcription factors *unbranched2* and *unbranched3* encoding SBP-box proteins also alter plant architecture and affect yield traits by regulating the rate of lateral primordia initiation [24].

MiRNAs are small non-coding 20–24 nt RNAs that can complementarily bind to their target mRNAs and reduce protein level through translational repression or transcript cleavage and degradation [25, 26]. Many development processes mediated by *SBP-box* genes are closely linked to miR156. Computational analysis indicated that many *SBP-box* genes are regulated by miR156 family in Arabidopsis [27]. Some important developmental processes seem to be mediated by both miR156 and their target *SBP-box* genes since overexpression of miR156 resulted in various phenotypes, including increased number of leaves, delayed flowering and decreased apical dominance [28]. Arabidopsis miR156 complementarily binds to the 3′UTR of *SPL3* mRNA and regulates its expression through translation inhibition and transcript cleavage [16, 29]. Overexpression of rice miR156 also resulted in decreased expression of the *SPL* target genes, suggesting the correlative interaction of *SPL* and miR156 in monocot plants [6]. Arabidopsis miR156 regulates tolerance to recurring heat stress and *SPL* genes are posttranscriptional regulated by miR156 after heat stress [30]. Recently, it is reported that miR156/SPLs modulates Arabidopsis lateral root development [31]. In addition to the regulatory roles of miR156, *SBP-box* genes were also shown to be regulated by miR529 in grasses [32]. Interestingly, miR156 and miR529 are correlated at the nucleotide level sharing a 14–16 nt binding site [33]. However, no miR529 candidates regulating *SBP-box* genes were found in core eudicots, such as Arabidopsis and poplar [34, 35].

Despite the essential roles of *SBP-box* genes in Arabidopsis or rice, information of *SBP-box* genes in oilseed rape (*B. napus*) is largely undefined. Genome-wide analysis of *SBP-box* genes has been performed in several species [36–40]. However, analysis of this gene family has not been conducted in *Brassica* species. Meanwhile, the interaction between the *BnaSBP* genes and *BnaMiR156* was not clearly understood. In the light of recent findings about *SBP-box* gene function in Arabidopsis, rice and other organisms, analysis of *SBP-box* genes in *B. napus* will certainly accelerate the utilization of these genes. Here we report the systematically analysis of *SBP-box* genes in *B. napus* for their gene structure, phylogeny, motif composition, miRNA target site, chromosomal localization and expression pattern in various tissues and organs. Moreover, the relative transcript level of *BnamiR156* in various tissues was also examined to study the functional relationship of *SBP* and *miR156* genes.

## Methods

### Identification and annotation of *SBP-box* genes in the *B. napus* genome

Firstly, the HMM profiles of the SBP domains (PF03110) in the Pfam database (http://pfam.xfam.org/) were downloaded and used to search the genome database of *B. napus* (http://www.genoscope.cns.fr/brassicanapus/) using HHMER search program. All non-redundant sequences were submitted to Interpro (http://www.ebi.ac.uk/interpro) to confirm the presence of the SBP domain. Sequences without complete SBP domain were excluded from the result. We also performed HHMER search against *Brassica rapa* and *Brassica oleracea* genome databases to identify SBP proteins. Secondly, Arabidopsis SBP protein sequences were downloaded from TAIR (http://www.*arabidopsis*.org/) to use as query to perform the BLASTP against *B. napus* genome. *SBP-box* gene accession numbers in *B. napus* genome database were extracted. The nomenclature of putative *SBP-box* genes in *B. napus* was in accordance with the homologous gene IDs in Arabidopsis. For one *SBP-box* gene in Arabidopsis, the orthologous *SBP-box* genes in oilseed rape were drawn up alphabetically. As the sequence of *AtSBP1* and *AtSBP12* shows high similarity, only *BnaSBP1* genes were named in oilseed rape. *SBP-box* genes in rice were downloaded from rice genome project (http://rice.plantbiology.msu.edu/).

### Gene structure, chromosomal location, duplication and phylogenetic analysis of *BnaSBP* genes

All the *BnaSBP* genes were mapped to the *B. napus* genome chromosomes according to the approximate position information. The exon/intron structure of each *BnaSBP* genes was displayed in Gene Structure Display Server program (http://gsds.cbi.pku.edu.cn/index.php) by comparing the coding sequence and genomic sequence. MCScanX software (http://chibba.pgml.uga.edu/mcscan2/) was used to analyze the duplication pattern of *BnaSBP* genes in oilseed rape genome. The local blast + software was used to perform the BLASTP analysis of *B. napus* with the e-value under 1e-5. The position of *SBP-box* genes and the blast output were imported into MCScanX software to generate a circle plot under a default criterion. Multiple sequence alignment of SBP-box protein sequence from *Oryza sativa*, *Arabidopsis thaliana* and *Brasscia napus* was performed using ClustalX2.0 with the default parameters [41]. Phylogenetic trees were constructed in MEGA6.0 software using the neighbor-joining (NJ) method and maximum likelihood (ML) method with 1000 bootstrap replications.

### Conserved motif identification and miR156 target site prediction

The conserved motifs were identified using the MEME online tool (http://meme-suite.org/) with parameter setup as following: maximum number of motifs, 20; number of repetitions, any; the range of motif width was from 6 to 80. All the identified motifs were searched in InterPro database (http://www.ebi.ac.uk/interpro/) and sequence logos were created using Weblogo online software (http://weblogo.threeplusone.com/). To predict the putative target sites of miR156, full length of *BnaSBP* genes including exon, intron and UTR sequences were analyzed using psRNATarget tool (http://plantgrn.noble.org/psRNATarget/?function). The conserved target sequences were modified by Genedoc software.

### Plant materials and growth condition

Plant samples used for expression pattern analysis and RNA-seq were collected from *B. napus* var. Zhongshuang 11 at the Oil Crops Research Institute of the Chinese Academy of Agricultural Sciences (OCRI-CAAS). The RNA-seq data were generated from twelve different tissues (root, leaf, bud, silique, stamen, new petal, blooming petal, wilting petal, stem, sepal, ovule and pericarp). The high resolution RNA-seq data of *BnaSBP* genes were kindly provided by Professor Shengyi Liu from OCRI-CAAS (data not published). The detailed FPKM value (Fragments Per Kilobase of exon model per Million mapped reads) was list in the supplemental data (Additional file 3: Table S2). The FPKM value was log2-transformed and the euclidean distances of all genes were calculated. Clustering tree was constructed and displayed by hierarchical cluster method of “complete linkage clustering” through *R* package.

To analyze the expression pattern of miR156 and *BnaSBP* genes, twelve tissue samples were also collected from the same tissue site at the same developmental stage as the sample for RNA-seq. All samples were collected and frozen in liquid nitrogen quickly and stored at the −80 °C. *B. napus* lines Purler and 6098B, harboring large and small branch angle respectively, were used for expression analysis. Results from different years showed that the branch angle of 6098B was 30−32° larger than that of Purler at the mature stage [42]. Tissue samples at the branch sites were collected at the bolting and early flowering stages for RNA-seq analysis. RNA-seq data were analyzed as described for Zhongshuang 11. Other tissue samples from 6098B and Purler were taken as those from Zhongshuang 11 to perform RT-PCR to verify the RNA-seq result. All plant materials were grown at the field in OCRI-CAAS, Wuhan, China.

### RNA extraction and quantitative real-time RT-PCR analysis

Total RNA from diverse tissues at different growth stage was extracted with Trizol Reagent (Invitrogen, America). Before reverse transcription, total RNA was treated with RNase-free DNase I (Promega, America) for 15 min to degrade genomic DNA. Stem-loop RT-PCR was used to examine miR156 expression level in different tissues following the procedure reported previously [43]. miRNA sequences in *B. napus* were downloaded from miRBase Sequence Database [44]. Primers used for stem-loop RT were designed according to Zhao et al. (2012) [45]. U6 specific primer was added simultaneously as reference for accurate normalization in each reaction. As the mature sequence of miR156 family varies in the 5′ region, five different forward primers were designed for realtime qPCR. qRT-PCR was run in CFX96 Real Time System (Bio-Rad, Hercules, California, USA) using SYBR Green (Tiangen, China) according to the instructions. Briefly, 12.5 μl SYBR mixture, 1 μl universal reverse primer and 1 μl specific primer were added for each reaction. The U6 reaction as a control was conducted using the specific primer. Three replicate reactions were performed for each sample using following program: 10 min at 95 °C, 40 cycles of 5 s at 95 °C, and 30 s at 60 °C. The specificity of the amplification for each primer pair was verified by melting curve analysis. For RT-PCR, two μg of RNA was used for first strand cDNA synthesis with a Transcript First Strand cDNA Synthesis Kit (Tiangen, China) according to manufacturer’s instructions. The reaction was conducted using following program: 5 min at 95 °C, 31–37 cycles of 30 s at 95 °C, 40 s at 54–60 °C and 1 min at 72 °C. Primers used in the qPCR and RT-PCR were listed in Additional file 1: Table S1. The *U6* and *actin* genes were selected as internal reference genes as described previously [45].

## Results

### Identification of SBP genes in *B. napus*

All Arabidopsis SBP protein sequences were used as queries for TBLASTN. As a result, fifty-eight putative *SBP-box* genes were identified initially. All the subsequences were checked by Interpro tool to search the SBP domain. Three proteins without SBP domain or with incomplete SBP domain were excluded. HHMER search was also performed against the *B. napus* protein database with SBP-domain PF03110 as a query. Ten additional protein sequences were obtained; however, only three of them contain the complete SBP domain checked by Interpro scan. Ultimately, fifty-eight SBP proteins were identified. Six SBP proteins could not be allocated at any *B. napus* chromosome accurately. All *SBP-box* genes in *B. napus* are designated as *BnaSBP* and named according to the order of closest orthologues in Arabidopsis. The accession number, chromosome distribution, protein molecular weight and length of the *BnaSBP* genes were listed in Table 1. HHMER search against *Brassica rapa* and *Brassica oleracea* genomes resulted in twenty-six and nineteen SBP proteins, respectively. Previous results have shown that sixteen SBP proteins exist in Arabidopsis. By comparison on number of genes in the three closely related species, *SBP-box* gene family members in *B. napus* showed an obvious expansion on number of genes.

**Table 1:** Nomenclature of *BnaSBP* genes

Gene name	Accession number ^a^	Length ^b^	MW (kd) ^c^	Introns	Locus ^d^
BnaSBP1a	BnaA05g00780D	869	96.83	11	-
BnaSBP1b	BnaC04g00420D	860	95.76	10	-
BnaSBP2a	BnaA06g36780D	519	57.61	4	+
BnaSBP2b	BnaC07g17030D	516	57.49	4	-
BnaSBP2c	BnaA09g16340D	390	43.67	4	-
BnaSBP2d	BnaC09g17430D	385	43.3	4	-
BnaSBP3a	BnaA05g09840D	142	16.63	1	-
BnaSBP3b	BnaC03g18800D	187	21.88	1	+
BnaSBP3c	BnaC04g44230D	141	16.49	1	+
BnaSBP3d	BnaA04g19840D	141	16.56	1	+
BnaSBP3e	BnaCnng05200D	147	17.01	1	-
BnaSBP4a	BnaC06g41420D	179	20.4	1	+
BnaSBP4b	BnaA06g01110D	161	18.48	2	-
BnaSBP4c	BnaA05g14670D	176	20.19	2	-
BnaSBP4d	BnaC06g10070D	157	18.11	3	-
BnaSBP5a	BnaC05g38350D	179	20.77	1	+
BnaSBP5b	BnaA05g24340D	179	20.73	1	+
BnaSBP5c	BnaA01g28740D	176	20.5	1	-
BnaSBP5d	BnaC01g36290D	176	20.56	1	+
BnaSBP6a	BnaA02g14580D	328	37.1	3	+
BnaSBP6b	BnaC02g19100D	333	37.88	2	+
BnaSBP6c	BnaC02g14000D	328	37.08	3	+
BnaSBP6d	BnaA07g27730D	299	33.98	0	+
BnaSBP6e	BnaCnng61400D	319	36.22	1	+
BnaSBP7a	BnaC02g08350D	778	87.04	9	+
BnaSBP7b	BnaC09g39030D	797	89.15	9	-
BnaSBP7c	BnaA10g16180D	794	89	9	-
BnaSBP7d	BnaCnng09040D	779	87.17	9	+
BnaSBP8a	BnaA10g00110D	312	34.82	3	+
BnaSBP8b	BnaC05g00110D	233	26.47	2	+
BnaSBP8c	BnaAnng08550D	335	37.18	2	+
BnaSBP9a	BnaC04g48150D	367	40.4	2	+
BnaSBP9b	BnaA05g02680D	368	40.03	2	+
BnaSBP9c	BnaC04g02520D	370	40.34	2	+
BnaSBP9d	BnaA04g24340D	363	39.71	2	+
BnaSBP10a	BnaA09g27950D	329	36.77	3	+
BnaSBP10b	BnaC07g11390D	372	41.54	4	-
BnaSBP10c	BnaC07g11380D	371	41.72	4	+
BnaSBP10d	BnaAnng25050D	346	38.88	5	+
BnaSBP11a	BnaC05g21280D	367	40.82	3	-
BnaSBP11b	BnaA07g08840D	390	43.65	3	-
BnaSBP11c	BnaA07g08830D	374	41.58	3	-
BnaSBP11d	BnaA09g27960D	365	40.66	3	-
BnaSBP11e	BnaC03g57620D	365	41.01	4	-
BnaSBP11f	BnaC05g21270D	364	40.73	3	+
BnaSBP13a	BnaC09g27080D	359	39.17	2	+
BnaSBP13b	BnaA03g13580D	341	37.35	2	+
BnaSBP13c	BnaC03g16490D	341	37.68	2	+
BnaSBP13d	BnaC03g27870D	348	38.2	2	+
BnaSBP14a	BnaC05g16270D	1032	114.16	9	+
BnaSBP14b	BnaA06g14810D	1031	114.01	9	+
BnaSBP14c	BnaC06g37430D	980	107.93	10	+
BnaSBP15a	BnaA07g17550D	316	35.25	2	-
BnaSBP15b	BnaC06g16200D	325	36.48	2	-
BnaSBP15c	BnaC04g23930D	324	36.13	2	+
BnaSBP15d	BnaA04g27550D	308	34.43	2	-
BnaSBP16a	BnaC02g24160D	1002	110.89	9	+
BnaSBP16b	BnaA07g32890D	960	105.92	11	+

^a^ Accession numbers was corresponded to the annotation provided by *Brassica napus* genome database

^b^ The AA length of BnaSBP protein

^c^ Molecular weight of BnaSBP protein

^d^ +, the sense strand; −, the antisense strand

### Chromosome localization and gene duplication analysis

**Fig. 1:**
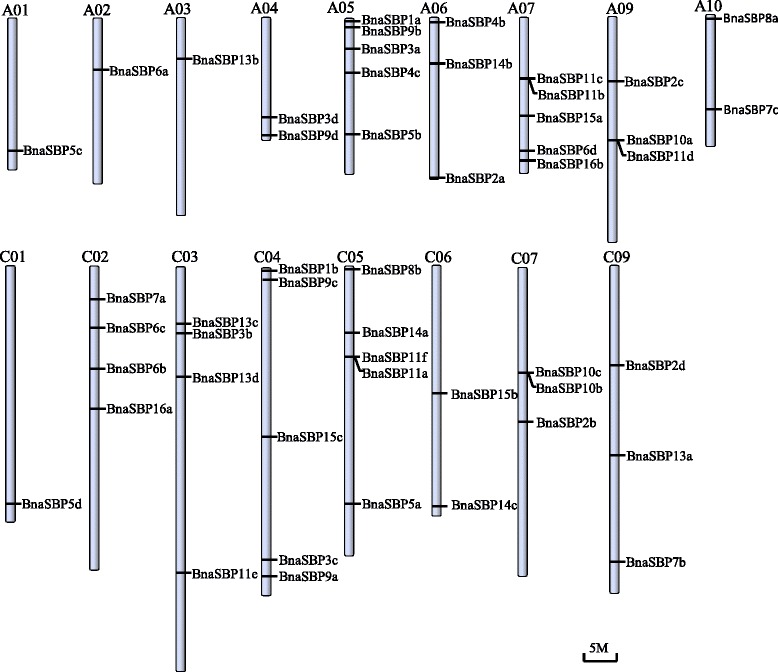
Distribution of *BnaSBP* genes on *B. napus* chromosomes numbered according to genome annotation database. Scale bar refers to a 5 Mb chromosomal distance

To determine chromosome distribution and gene duplication of *SBP* genes in *B. napus*, all the *SBP* genes except four located on unanchored scaffolds, were mapped to approximate chromosome positions (Fig. 1). These fifty-four *SBP* genes were unevenly distributed on the *Brassica* chromosomes. Except for A8 and C8, all chromosomes harbor at least one of the *SBP* genes. On chromosome A1, A3 and C1, only one *SBP* gene was found. Four chromosomes contain the maximum number of *SBP* genes, i.e., A5, A7, C4 and C5 each has five *SBP* genes. Four clusters each with two *SBP* genes were identified by the criteria that the distance of adjacent *SBP* genes is less than 50 kb. Twenty-six and thirty-two *SBP* genes were found to located at the A genome and C genome respectively. It was interesting to find that the number of *SBP* genes located at the A genome of *B. napus* was equal to the number of *SBP* gene found in the *B. rapa* genome. However, only 19 *SBP* genes were identified in *B. oleracea* genome, which is the progenitor of the C genome in *B. napus*, indicating that the *SBP* gene expansion may have occurred in the polyploid C genome.

**Fig. 2:**
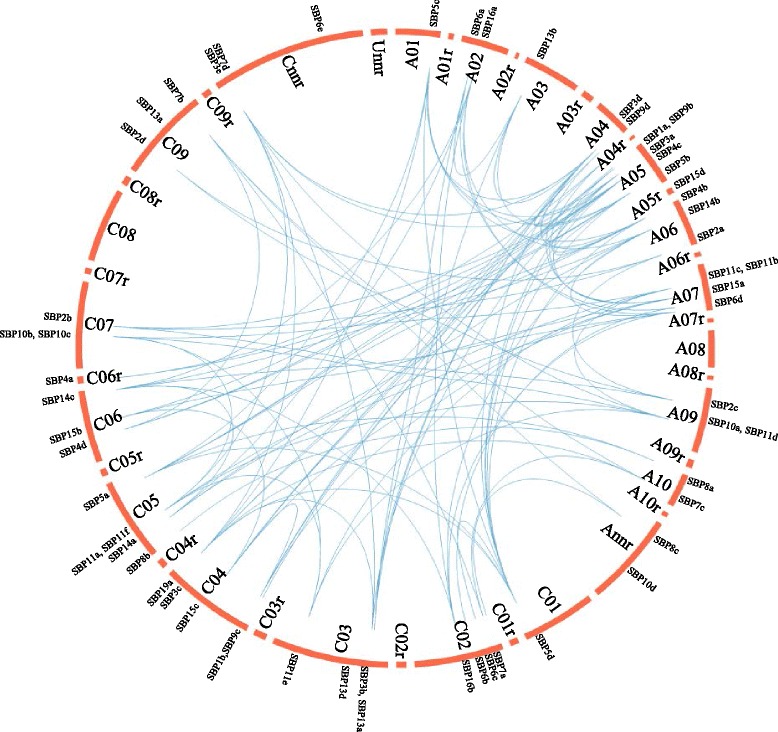
Circle plot showing segmental duplication of *BnaSBP* genes on 19 *B. napus* chromosomes. Blue lines indicated duplication of *BnaSBP* genes

The tandem and segmental duplication of *Brassica SBP* genes were also analyzed. Among all the *SBP* genes, eight members (13.8 %) showed tandem repeats, which include four clusters of tandem repeat genes (Fig. 1). In addition, 49 (84.5 %) of the fifty-eight *BnaSBP* genes were found to be segmentally duplicated genes. These genes were located at seventeen different chromosomes (Fig. 2).

### Structural organization and conserved domain identification

**Fig. 3:**
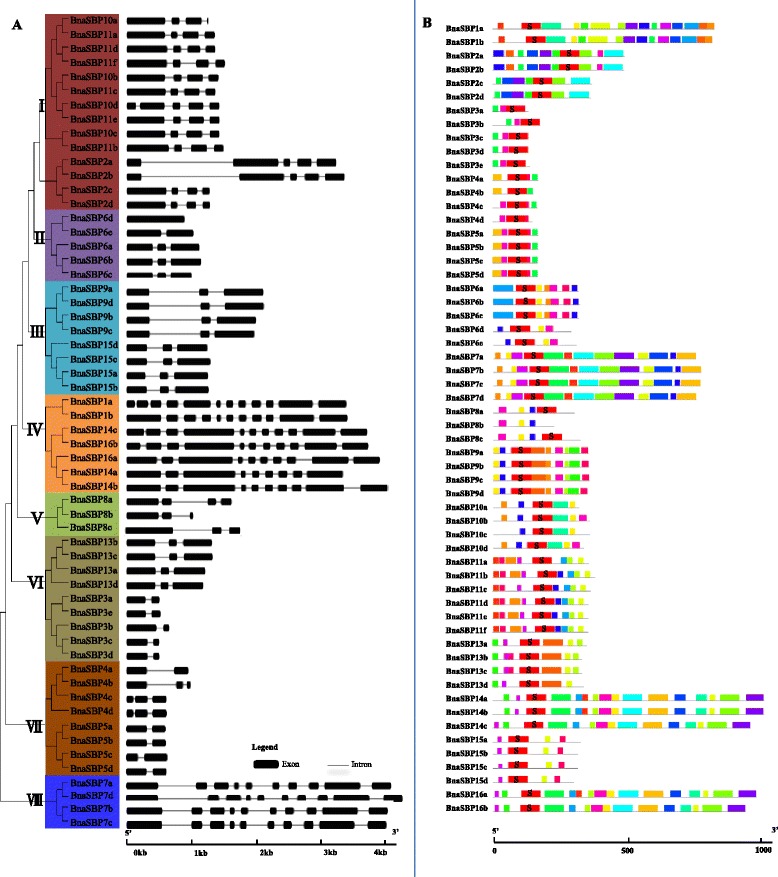
Phylogenetic relationship and gene structure of *SBP-box* genes in *B. napus*. **a** Unrooted phylogenetics tree and structures of *SBP-box* genes. Unrooted phylogenetic tree was created in MEGA6 software with the neighbor-joining method with 1000 bootstrap iterations according to the 58 coding sequence of *SBP-box* genes. Exons and introns were represented by boxes and lines, respectively. Size of exons and introns can be estimated using the scale bar at bottom. **b** Motif prediction of BnaSBP proteins. Twenty motifs were identified by MEME online tool. Each motif is represented by a colored block. S represents the SBP domain. The length and position of the motifs could be estimated according to the scale bar

To understand the evolutionary relationship among SBP protein in *B. napus*, we constructed the unrooted tree based on the alignments of full-length SBP protein sequences using neighbor-joining (NJ) method in MEGA 6.0. The fifty-eight SBP proteins in *B. napus* were divided into eight distinct groups (from Ito VII). Group I consist of the maximum number (14) of BnaSBPs, while group Vcontains only three BnaSBPs. The entire tandem duplicated BnaSBPs were assigned to one group, in accordance with the results reported in other species, such as tomato, *Populus trichocarpa* [40, 46]. The genomic sequence of the *BnaSBP* genes ranged from 510 bp to about 5 kb. To obtain further gene structure information, we compared the coding sequence with the genomic sequence of all *BnaSBP* genes (Fig. 3a). Different introns (from 0 to 10) were observed among the *BnaSBP* genes. Except *BnaSBP6d*, all *BnaSBP* genes contain at least one intron. The genes possess maximum number of introns were in group IV and VII. The *BnaSBP* gene clusters that were divided into the same group exhibited similar structure. Several motifs were identified among SBP proteins in *B. napus* (Fig. 3b). One motif (S) containing the SBP-domain was detected in all BnaSBP proteins except BnaSBP8b which contains a similar SBP-domain that could not be detected due to missing of a few amino acids. The BnaSBP protein in the same group exhibited similar motif composition.

**Fig. 4:**
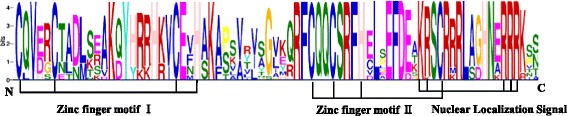
Sequence logo of the *B. napus* SBP-box domain. Multiple sequence alignment was performed by using clustalW2. Sequence logo was obtained from Weblogo online software. The *X-axis* represents the conserved sequences of the SBP domain. The overall height of letters represents residue conservation. The *Y-axis* reflects the conservation rate of each amino acid. Two zinc finger and one nuclear localization signal motifs are indicated

All the BnaSBP proteins were aligned by the ClustalX 2.0 and the conserved SBP domain was created by the Weblogo online tools. Fifty-eight BnaSBP proteins contained the complete SBP domain with two Zinc motifs and one nuclear localization signal (Fig. 4). The first zinc finger motif was C3H type in all the SBP proteins except BnaSBP5 group. All the SBP proteins contain the second CCHC type zinc motif. As SBP proteins possess the character of transcription factors, all the SBP proteins contain the conserved nuclear localization signal.

### Phylogenetic analysis of *SBP* genes in *B. napus*, Arabidopsis and rice

**Fig. 5:**
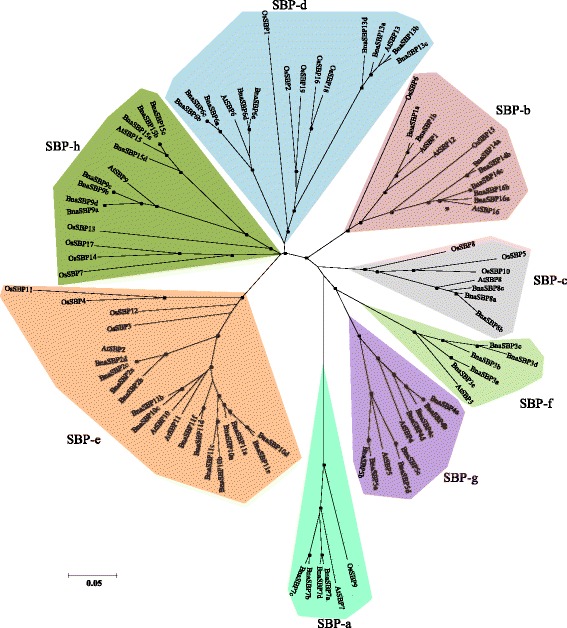
Phylogenetic analysis of BnaSBP proteins. The protein sequences of SBP-box from Arabidopsis (AtSBP), rice (OsSBP) and *B. napus* (BnaSBP) were aligned using ClustalW. The phylogenetic tree was constructed using the neighbor-joining algorithm with 1000 replications. Nodes with bootstrap values of >50 % are dotted. Bar indicates 0.05 aa substitution per residue

The phylogenetic relationship among *BnaSBP* genes and other *SBP* genes with known functions from other species is useful for predicting their roles in oilseed rape development. Sixteen *SBP* genes from Arabidopsis and nineteen *SBP* genes from rice, which are model plants for dicot and monocot species respectively, were extracted from the public gene pool. Fifty-eight *SBP* genes from oilseed rape together with the Arabidopsis and rice genes were used for the construction of an unrooted phylogenetic tree (Fig. 5, Additional file 2: Figure S2). According to phylogenetic analysis, *SBP* genes from these three plant species can be classified into seven groups (SBP-a to SBP-h). The largest group (SBP-e) contains 21 members which account for 23 % of the total SBPs, whereas group SBP-a forms the smallest group containing only five members. As shown in Fig. 5, genes in group SBP-a were more diverged than those in other groups. *BnaSBP* genes showed a high similarity to their orthologs from Arabidopsis and were classified into the same group. Among the groups revealed by phylogenetic analysis, group SBP-f only contain SBPs from Arabidopsis and oilseed rape, indicating the diversification of *SBP* genes between monocot and dicot plants.

### MiR156 family in *B. napus* and their target site to *BnaSBP* genes

**Fig. 6:**
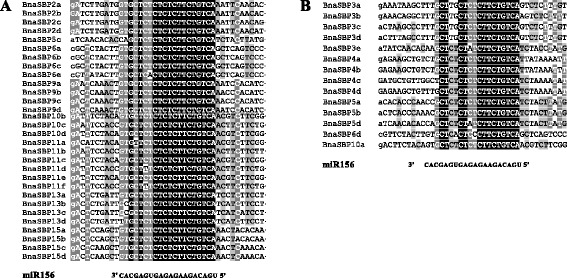
Sequence alignment of miR156 complementary sequences of the *BnaSBP* genes. **a** The complementary sequences are located in the coding regions. **b** The complementary sequences are located in the 3′UTR regions

Seven putative members of miR156 (BnaMiR156a-g) in oilseed rape were found after querying the miRBase database. Recently, thirty-two putative pre-mature structures of miR156 were predicted in *B. napus* by high throughput small RNA deep sequencing [47]. Previous results showed that miR156 complementarily bind to *SBP* genes either at the coding or 3′UTR region and reduced gene activity by translation suppression or cleavage [27, 29]. It was shown that 44 SBP proteins have miR156 binding site, with 30 and 14 at coding and 3′UTR regions, respectively (Fig. 6). According to previous results, 11 out of 17 *SBP* genes in Arabidopsis are targeted by miR156. The homologous genes in oilseed rape are also predicted to be target of miR156. These results suggest that relationship between miR156 and *SBP* genes is conserved across species. However, three *BnaSBP* genes targeted by miR156 differed from other genes. *BnaSBP5c* possesses the binding site within the coding region, while the other three *BnaSBP5* genes are targeted by miR156 in 3′UTR. MiR156 was predicted to bind to 3′UTR sequence of *BnaSBP6d* and *BnaSBP10a*, while the relative homologous gene in Arabidopsis were bound by miR156 at the coding region. The distinct regulation pattern of the homologous genes between *B. napus* and Arabidopsis reveals the divergence of the SBP-box genes in oilseed rape.

### Expression profile of *BnaSBP*

**Fig. 7:**
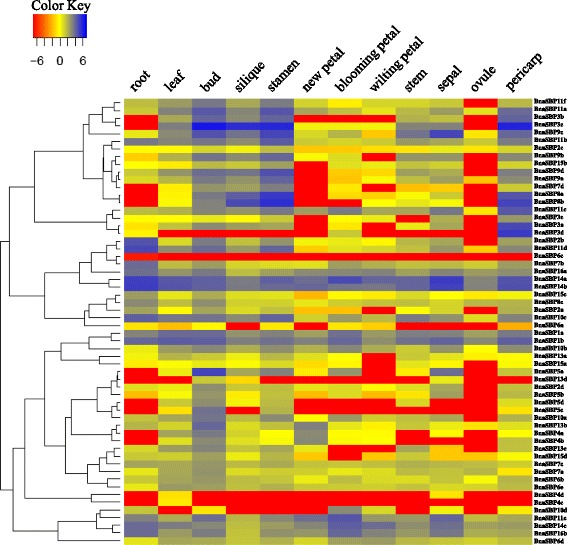
Expression patterns of *BnaSBP* genes in twelve different tissue samples. *Color scale bar* at the top of map represents log2 transformed FPKM values, which represents low and high expression, respectively. Tissues used for expression profiling are indicated at the top of each column. The genes are on right of expression bar

**Fig. 8:**
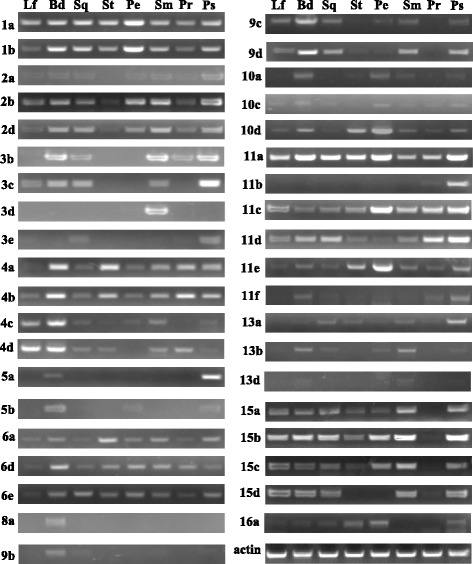
Analysis expression level of *BnaSBP* genes in eight different tissue samples of Zhongshuang 11 by RT-PCR. Tissues used for expression profiling are indicated at the top of each column. Lf, leaf; Bd, bud; Sq, silique; St, Stamen; Pe, petal; Sm, stem; Pr, pericarp; Ps, pistil. The genes are on left of expression bar. *Actin*, *BnaSBP11e* and 2b gene was amplified with 28, 31 and 37 cycles respectively. Other *BnaSBPs* genes were amplified with 35 cycles

A wide range of *SBP* genes play important roles in plant development process. In the absence of *SBP* gene mutants, the expression pattern may provide a clue to elucidate the potential role of the different *SBP* genes in *B. napus*. The expression level of *BnaSBP* genes in twelve tissues were shown by heat map representation (Fig. 7, Additional file 3: Table S2). Transcript of *BnaSBP6c* was zero in all twelve tissue samples and only very low expression level of *BnaSBP4c* in leaf was detected. Based on the hierarchical clustering analysis, the *BnaSBP* genes could be divided into eight categories. The transcription of a large number of *BnaSBP* genes was enriched in bud, stamen and pericarp. By contrast, most of *BnaSBP* genes exhibit low expression level in ovule and petal. Eight *BnaSBP* genes, *BnaSBP1a*, *1b*, *11e*, *14a*, *14b*, *14c*, *16a* and *16b* seemed to be expressed constitutively, from root to pericarp. It should be noted that all these genes, excluding *BnaSBP11e*, are not predicted to be targeted by the miR156. *BnaSBP4c*, *4d*, *5c*, *5d*, *10d* and *13d* sustained low expression level in most tissues. The expression level of *BnaSBP3a* and *3d* was not detected in most tissue samples, but reached clearly higher levels in pericarp. A relative higher expression level of *BnaSBP2b* and *11d* could also be discerned in root tissue. Compared with the *SBP* genes not bound by miRNA, the *BnaSBP* genes have the target site represent more divergent expression pattern. We also performed RT-PCR to confirm the expression levels of some *BnaSBP*s in eight different tissues (Fig. 8). Thirty-nine *BnaSBP*s were selected to verify the result of RNA-seq data. Results showed that RT-PCR data was generally consistent with RNA-seq data for relative expression of *BnaSBP*s in most of the tissues. For example, expression level of *BnaSBP1a*, *1b* and *11e* could be detected in most tissues (Fig. 8). Though *BnaSBP*s were expressed at least in one of the tissues, distinction of expression patterns were observed across the gene groups. Some *BnaSBP*s belongs to a same group exhibited similar expression pattern, such as *BnaSBP1a* and *1b* in group IV, *BnaSBP15a* and *15b* in group III, indicating redundant roles of *BnaSBP*s in the same group. Therefore, the oilseed rape SBP transcription factors have diverse expression patterns and may be redundant in biological function with each individual in charge of certain physiological processes.

**Fig. 9:**
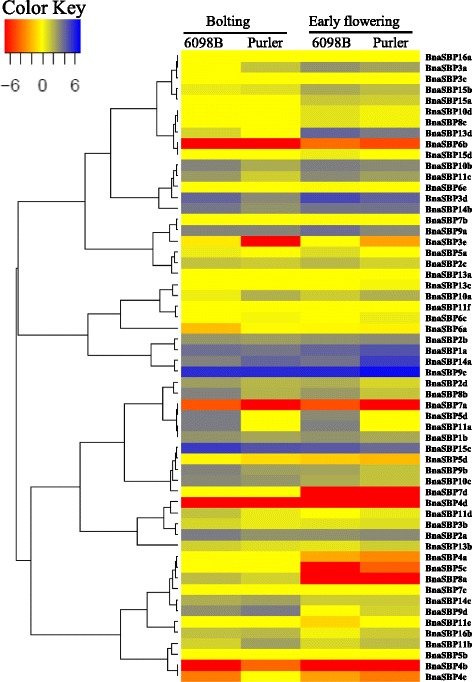
Expression patterns of *BnaSBP* genes in the branching site of 6098B and Purler at the bolting and early flowering stages. *Color scale bar* at the top of heat map represents log2 transformed FPKM value, which represent low and high expression, respectively. Tissues used for expression profiling are indicated at the top of each column. The genes are on right of expression bar

**Fig. 10:**
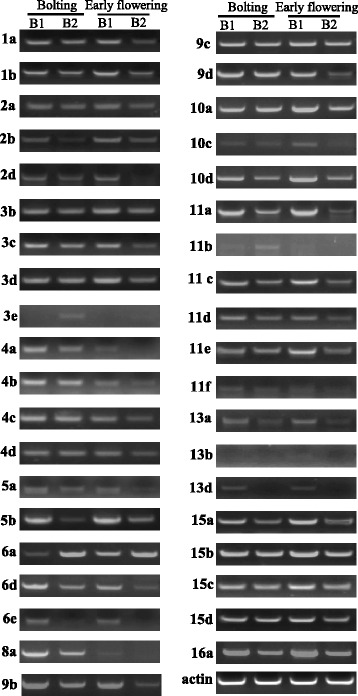
Expression level of *BnaSBP* genes in the branching site of 6098B and Purler at the bolting and early flowering stages by RT-PCR. Tissues used for expression profiling are indicated at the top of each column. B1, branch site in 6098B; B2, branch site in Purler. Bolting and early flowering time represent sampling time. The genes are on left of expression bar. *Actin*, *BnaSBP11e* and *2b* gene was amplified with 28, 31 and 37 cycles respectively. Other *BnaSBPs* genes were amplified with 35 cycles

To investigate the putative genes involved in branch angle regulation, the expression profile of two *B. napus* material (6098B and Purler) with different branch angle was conducted (Additional file 4: Figure S1). Sample of branch site from two materials at bolting and early flowering stage was harvested to perform DEGs (Different Expression Genes). The transcription level of all *SBP* genes was extracted from expression profile (Additional file 5: Table S3). Heat maps representing expression levels in the lines at two developmental stages are shown in Fig. 8. Many *BnaSBP* genes showed different expression patterns between the two lines at the two development stages. *BnaSBP5c*, *8a* and *7d* showed high expression at bolting stage but no or little expression at early flowering stage in the two materials. Ten and thirteen *BnaSBP* genes were found differentially expressed between the two lines at the two development stages, respectively. Among them, six *BnaSBP* genes were differentially expressed at the two development stages (Fig. 9). Further studies may focus on the role of these genes on branch angle regulation. RT-PCR was performed to confirm the expression level of *BnaSBP*s in the same tissues used for RNA-seq. A large number of *BnaSBP*s in Purler expressed at higher level than those in 6098B (Fig. 10). This RT-PCR result was generally consistent with that from RNA-seq data.

### Expression profile of miR156

**Fig. 11:**
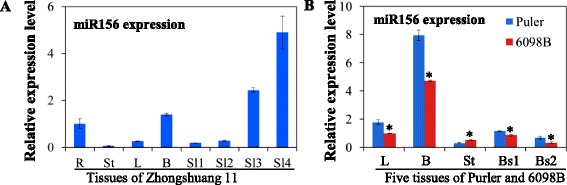
The expression patterns of miR156 in different tissue samples. Relative expression levels of mature miR156 in different tissues were analyzed by qRT-PCR. **a** The expression level of miR156 in the different tissue samples of Zhongshuang11. The value was normalized to the root at the seedling stage. R, root; St, stem; L, leaf; B, bud; Sl1, silique 15 days after flowering; Sl2, silique 18 days after flowering; Sl3, silique 20 days after flowering; Sl4, silique 23 days after flowering. **b** The expression level of miR156 in five tissue samples of Puler and 6098B respectively. The value was normalized to 6098B at the bolting stage. L, leaf; B, bud; St, stem; Bs1, branch site at the bolting stage; Bs2, branch site at the early flowering stage. Asterisks indicate a significant difference was detected between Purler and 6098B in the same tissue sample by *t-test* at *P < 0.01

Several *BnaSBP* genes carry the complementary sequences to miR156. MiR156 was thus expected to be an important determinant for the expression of these *BnaSBP* genes. The expression level of miR156 was mostly abundant in bud and silique of Zhongshuang 11 at different developmental stages (Fig. 11a). Relative low levels were found in leaf sample. Meanwhile, the expression level of miR156 in 6098B and Purler was also determined. It was showed that the abundance of miR156 decreased significantly at early flowering time compared to bolting time (Fig. 11b). Besides the stem sample of two materials, the transcription of miR156 was stronger in Purler than in 6098B of the other tissues.

## Discussion

### *SBP-box* genes in *Brasscia* and their evolution

The SBP-box proteins are characterized by a conserved SBP domain with 76 amino acids and constitute one large family of transcription factors in plants. Plant specific SBP-box transcription factors were only detected in green plants suggesting that it might originate predating the divergence of green algae and the ancestor of land plants [5, 48]. Different numbers of *SBP-box* genes have been characterized in various land plants [39, 40, 49]. In present study, 58 *SBP-box* genes in *B. napus* genome were identified, which is about four times the number of Arabidopsis *SBP-box* genes. *B. napus* contains 13 more *SBP-box* genes than the sum of *B. rapa* (AA, 2n = 20) and *B. oleracea* (CC, 2n = 18), which are two immediate progenitor species of *B. napus* (AACC, 2n = 38) [50]. For one gene family, tandem and segmental duplication events are the main reasons for gene expansion. *SBP-box* genes are unevenly distributed on 17 of the 19 chromosomes of *B. napus*, and four clusters each with two *BnaSBP*s were identified (Fig. 1). Uneven and cluster distribution of *SBP-box* gene family genes was also found in rice and peach [6]. There are seven and 49 *BnaSBP* genes which were found to be tandem and segmental duplications respectively. Diversification of *BnaSBP* genes was observed from many aspects, including phylogenesis, genomic structure, as well as location of miR156 target site. This diversity of *SBP-box* gene structure is likely to be trigged by gene duplication followed by intron and exon loss.

### Functional divergence of *SBP-box* genes

As the *SBP-box* genes possess the character of transcription factors, their expression pattern is expected to be correlated with their function on plant development. The expression profile of *BnaSBP-box* genes showed distinct expression patterns among different tissues. In Arabidopsis, some *SPL* genes are constitutively expressed, while the transcription level of others is under developmental control [5]. Expression analysis of *SBP-box* genes in other organisms also presented diverse spatiotemporal expression patterns [39, 40, 49, 51]. SBP transcription factors in *B. napus* showed diverse expression patterns across tissues, indicating their possible functions in various biological processes. The transcription of a large number of *BnaSBP* genes was enriched in bud, stamen and pericarp, suggesting most of the *SBP-box* genes in oilseed rape may be involved in the development of reproductive organs.

*SBP-box* genes in many species, especially in rice and Arabidopsis, have been demonstrated to play essential roles in diverse developmental processes. The microRNA regulated *SBP-box* genes *SPL9* and *SPL15*, which are the most close orthologous genes in Arabidopsis, was proven to control shoot maturation [52]. Further support of possible roles for *BnaSBP* in development comes from the rice genes *SPL14* in panicle development and ideal rice plant architecture regulation [22, 23]. We identified four *BnaSBP*9 genes in oilseed rape genome. Although the *BnaSBP9* genes possess similar gene structure, diverse expression patterns were observed. It should be noted that the expression of *BnaSBP9d* in the compact material Purler is higher than in the loose material 6098B (Figs. 9 and 10). The expression of *BnaSBP9d* visibly decreased from bolting to early flowering. Further study should be performed to verify whether *BnaSBP9d* might play a role in regulating branch angle in oilseed rape.

Arabidopsis gene *SPL8* affects pollen sac development and also controls gynoecium patterning [18]. Three *BnaSBP* genes, *BnaSBP8a*, *8b* and *8c* showed most similarity to *AtSBP8*,joining the same group through phylogenetic analysis. *BnaSBP8a* and *BnaSBP8b* were highly expressed in the stamen. Further study may focus on the potential role of *BnaSBP8* in flower development.

Constitutive expression of *AtSPL3* resulted in early flowering [53]. The *SPL3* homologous genes in *Antirrhinum majus* and *Silver birch* also regulate flower development by binding to the MADS-box genes [16, 54]. Tomato *LeSPL-CNR*, which is most similar to *AtSPL3* gene, is crucial for normal fruit development and ripening [55]. In Arabidopsis, miR156-SPL3 module controls FT expression to regulate ambient temperature-responsive flowering [56]. Among the five genes homologous to *AtSPL3* identified in *B. napus* in our study, *BnaSBP3c* showed much higher expression level in bud, stamen, silique and pericarp, indicating a possible role in the reproduction phase. Arabidopsis gene *AtSPL2*, *AtSPL10* and *AtSPL11* were shown to play important roles in determining leaf shape and embryonic morphogenesis [20, 57]. All the *BnaSBP2*, *10* and *11* genes were classified into a same group of SBP-e. It would be interesting to explore the exact role of these group *SBP-box* genes by functional characterization.

### Conservation of miR156 target site in *SBP-box* genes

A larger number of miRNAs targets are transcription factors, such as SBP, MYB, NAC, ARF, GRAS, and AP2 [27]. MiRNAs play important roles in regulating the transcription of target genes. Previous results showed that overexpression of miR164, miR159a, and miR319 affected members of the NAC, MYB, and TCP families of transcription factor genes, respectively [58–60]. In present study, target prediction showed that 44 of the 58 *BnaSBP* genes were regulated by miR156. The complementary sites of miR156 locate in the coding region of 30 *BnaSBP* genes, and in the 3′ UTR of the other 14 *BnaSBP* genes. In Arabidopsis, 10 (*AtSBP2, 3, 4, 5, 6, 9, 10, 11, 13, 15*) out of 17 *SBP* genes were predicted or verified to be targeted by miR156. The other six At*SBP* genes including (*AtSBP1, 7, 8, 12, 14, 16*) are not targets of miR156. *AtSPL7* has been demonstrated to bind directly to the Cu-response element (CuRE) containing a core sequence of GTAC and regulate Cu homeostasis [3]. The 44 *BnaSBP* genes predicted to be targeted by miR156 are the homologous genes in Arabidopsis, which also formed 10 gene clusters. Therefore, the miR156 target site in SBP-box genes is conserved across plant species.

Over-expression of miR156 in Arabidopsis significantly represses the *SPL* transcription and thus reduces apical dominance, leading to dwarfism and increases in total leaf number and plant biomass [28]. The transcripts of the target *SBP* genes were also suppressed in other miR156 over-expression plants [29, 56]. In present study, the transcript level of miR156 was abundant in bud and silique (Fig. 11). By contrast, most putative target *SBP* genes with predicted miR156 target sites showed lower expression level in these tissues (Figs. 9 and 10). Among the floral organs, most *BnaSBP* genes showed a low expression level in petal and ovule, though transcript was relatively high in pericarp, which is a main component of silique. These results suggested that the transcript of miR156 is negatively correlated with the expression of most *BnaSBP* genes. The level of miR156 was declined with a concomitant rise in *SPL* levels during the aging time in Arabidopsis [61]. *SPL9* and *SPL10* mediated the transition from high levels of miR156 to high levels of miR172 through direct activation of miR172 expression, thereby promoting the juvenile to adult phase transition [57, 62]. Our results showed that the lower expression level of miR156 in 6098B with bigger branch angle than in Purler with smaller branch angle (Fig. 11) is negatively correlated with the expression difference of many *SBP-box* genes, eg. *BnaSBP2a, 2d, 3d, 3e, 5d, 8b, 9a, 9b, 10b, 11a, 11c, 13d* and *15c* (Figs. 9 and 10), indicating that the SBP/miR156 module is likely involved in regulating plant architecture in *B. napus*.

## Conclusion

By genome wide analysis of *SBP-box* genes in oilseed rape (*B. napus L*), 58 *SBP-box* genes were identified in the *B. napus* genome. The BnaSBP proteins were classified into eight different groups and showed clear orthologous relationships of SBP members from rice and Arabidopsis. Our results showed that many *SBP-box* genes, which were predicted to be targeted by miR156, have tissue specific expression pattern and the expression pattern diverged after gene duplication. The expression level of miR156s was abundant in the root, flowers and silique samples. The different expression pattern between the miR156 and *SBP-box* genes in diverse tissues suggests that SBP/miR156 module may play an important role in the development processes. Eleven *SBP-box* gene groups, similar to those in Arabidopsis, were predicted to be targeted by miR156, implying the conservation of SBP/miR156 module regulation pattern. The involvement of some *BnaSBP* genes as well as the SBP/miR156 module in plant architecture regulation was also implicated from the results. Taken together, our data presented here provide valuable information for further study on the function of *SBP-box* in *B. napus*.

## Abbreviations

CuRE, Cu-response element; Mw, The molecular weight; SBP, squamosa promoter binding protein

## Additional files

Additional file 1: Table S1. Primers used for quantitative polymerase chain reaction (qPCR) in gene expression analysis. (DOC 36 kb) Additional file 2: Figure S2. Phylogenetic analysis of BnaSBP proteins. The conserved SBP domain sequences encoded by Arabidopsis (AtSBP), rice (OsSBP) and *B. napus* SBP-box proteins were aligned using ClustalW. The phylogenetic tree was constructed using the maximum likelihood method with 1000 replication. Bar indicates 0.1 aa substitution per residue. (PPTX 73 kb) Additional file 3: Table S2. Absolute gene expression values in twelve tissue samples. (XLSX 18 kb) Additional file 4: Figure S1. Phenotypes of two lines with different branch angle. (A) 6098B and Purler lines grown at the middle flowering stage. Bar = 25 cm. (B) The branch angle of 6098B is larger than that of Purler. The arrows indicate the different branch angle of two lines. Bar = 2 cm. (PPTX 145 kb) Additional file 5: Table S3. Absolute gene expression values in two samples. (XLSX 15 kb)
