# Supplementary material for: Genomic identification, characterization and differential expression analysis of SBP-box gene family in Brassica napus
Source: BMC Plant Biol. 2016 Sep 8;16(1):196. doi: 10.1186/s12870-016-0852-y (PMC5017063; doi:10.1186/s12870-016-0852-y)
Supplement: Additional file 1: Table S1. — Primers used for quantitative polymerase chain reaction (qPCR) in gene expression analysis. (DOC 36 kb) [file 12870_2016_852_MOESM1_ESM.doc]

**Additional file 1: Table S1.** Primers used for quantitative polymerase chain reaction (qPCR) in gene expression analysis

| Primer name | Primer sequence | Use |
| --- | --- | --- |
| 156RTR1 | GTCGTATCCAGTGCAGGGTCCGAGGTATTCGCACTGGATACGACGTGCTC | Reverse transcription for miR156 genes |
| MIRQ | GTGCAGGGTCCGAGGTATTC | Universal reverse primer for real-time PCR |
| 156QF | AGCAGCCATGACAGAAGAGAGT | Amplifying miR156 gene for real-time PCR |
| U6s | TTGGAACGATACAGAGAAGATTAGCA | Amplifying U6 gene for real-time PCR |
| U6a | TTGGACCATTTCTCGATTTGTG | Reverse transcription and amplifying U6 gene in real-time PCR |
