# Supplementary material for: Genomic identification, characterization and differential expression analysis of SBP-box gene family in Brassica napus
Source: BMC Plant Biol. 2016 Sep 8;16(1):196. doi: 10.1186/s12870-016-0852-y (PMC5017063; doi:10.1186/s12870-016-0852-y)
Supplement: Additional file 2: Figure S2. — Phylogenetic analysis of BnaSBP proteins. The conserved SBP domain sequences encoded by Arabidopsis (AtSBP), rice (OsSBP) and B. napus SBP-box proteins were aligned using ClustalW. The phylogenetic tree was constructed using the maximum likelihood method with 1000 replication. Bar indicates 0.1 aa substitution per residue. (PPTX 73 kb) [file 12870_2016_852_MOESM2_ESM.pptx]

## Slide 1
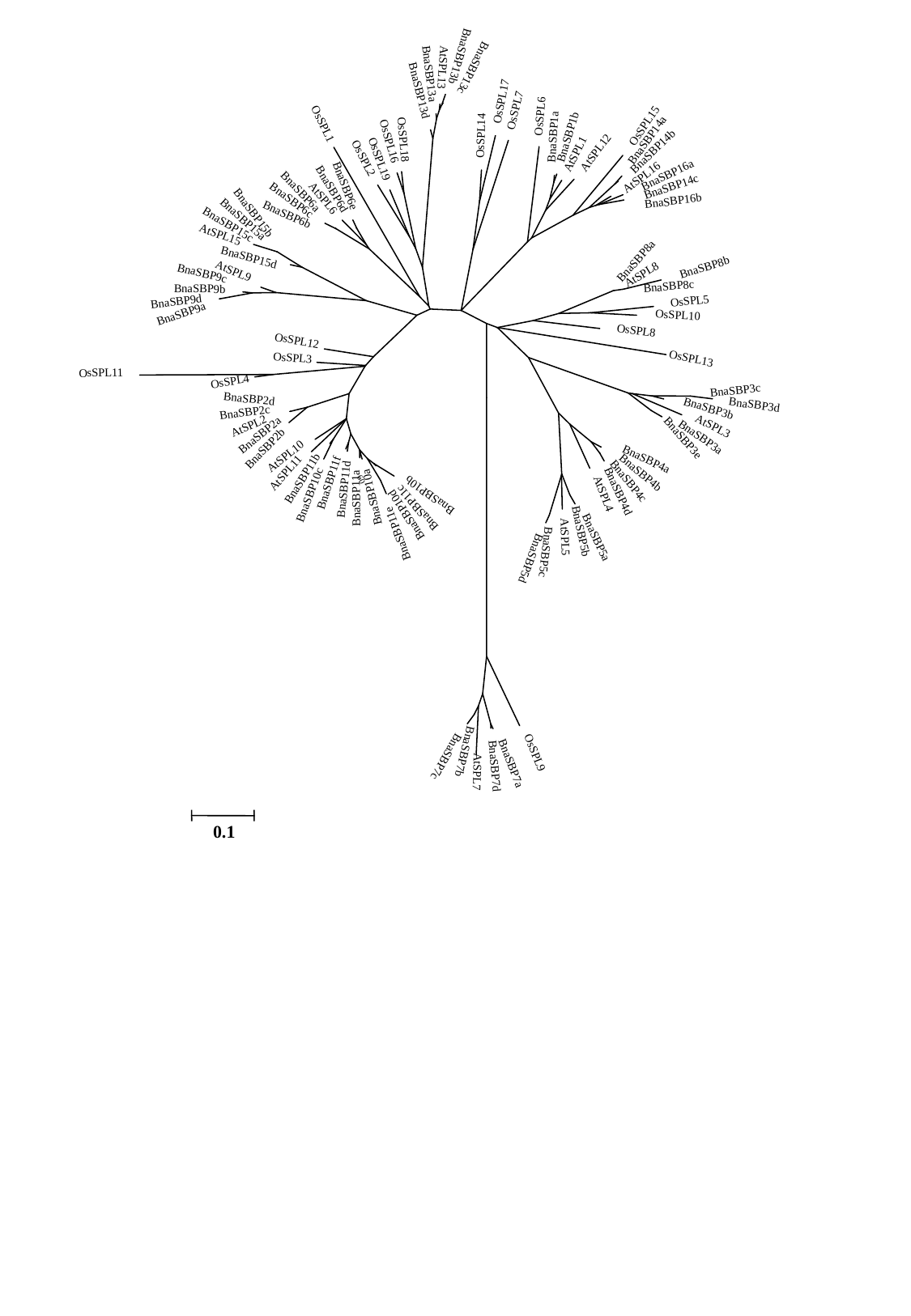

BnaSBP13b
AtSPL13
BnaSBP13c
BnaSBP13a
BnaSBP13d
OsSPL17
OsSPL7
OsSPL6
OsSPL1
OsSPL15
OsSPL14
BnaSBP1b
BnaSBP1a
BnaSBP14a
OsSPL18
OsSPL16
BnaSBP14b
AtSPL12
AtSPL1
OsSPL2
OsSPL19
BnaSBP16a
AtSPL16
BnaSBP6e
BnaSBP14c
BnaSBP6d
BnaSBP6a
AtSPL6
BnaSBP6c
BnaSBP16b
BnaSBP15b
BnaSBP6b
BnaSBP15a
BnaSBP15c
AtSPL15
BnaSBP15d
BnaSBP8a
BnaSBP8b
AtSPL9
BnaSBP9c
AtSPL8
BnaSBP8c
BnaSBP9b
OsSPL5
BnaSBP9d
BnaSBP9a
OsSPL10
OsSPL8
OsSPL12
OsSPL3
OsSPL13
OsSPL11
OsSPL4
BnaSBP3c
BnaSBP2d
BnaSBP3d
BnaSBP3b
BnaSBP2c
AtSPL2
AtSPL3
BnaSBP2a
BnaSBP3a
BnaSBP3e
BnaSBP2b
AtSPL10
BnaSBP4a
BnaSBP4b
AtSPL11
BnaSBP11b
BnaSBP4c
60
BnaSBP11f
BnaSBP11d
BnaSBP4d
AtSPL4
BnaSBP10c
BnaSBP10b
BnaSBP10a
BnaSBP11a
BnaSBP11c
BnaSBP10d
BnaSBP5b
BnaSBP11e
AtSPL5
BnaSBP5a
BnaSBP5c
BnaSBP5d
BnaSBP7b
OsSPL9
BnaSBP7c
BnaSBP7a
BnaSBP7d
AtSPL7
0.1
