# Supplementary figures and images for: Genomic identification, characterization and differential expression analysis of SBP-box gene family in Brassica napus
Source: BMC Plant Biol. 2016 Sep 8;16(1):196. doi: 10.1186/s12870-016-0852-y (PMC5017063; doi:10.1186/s12870-016-0852-y)

## Slide 1
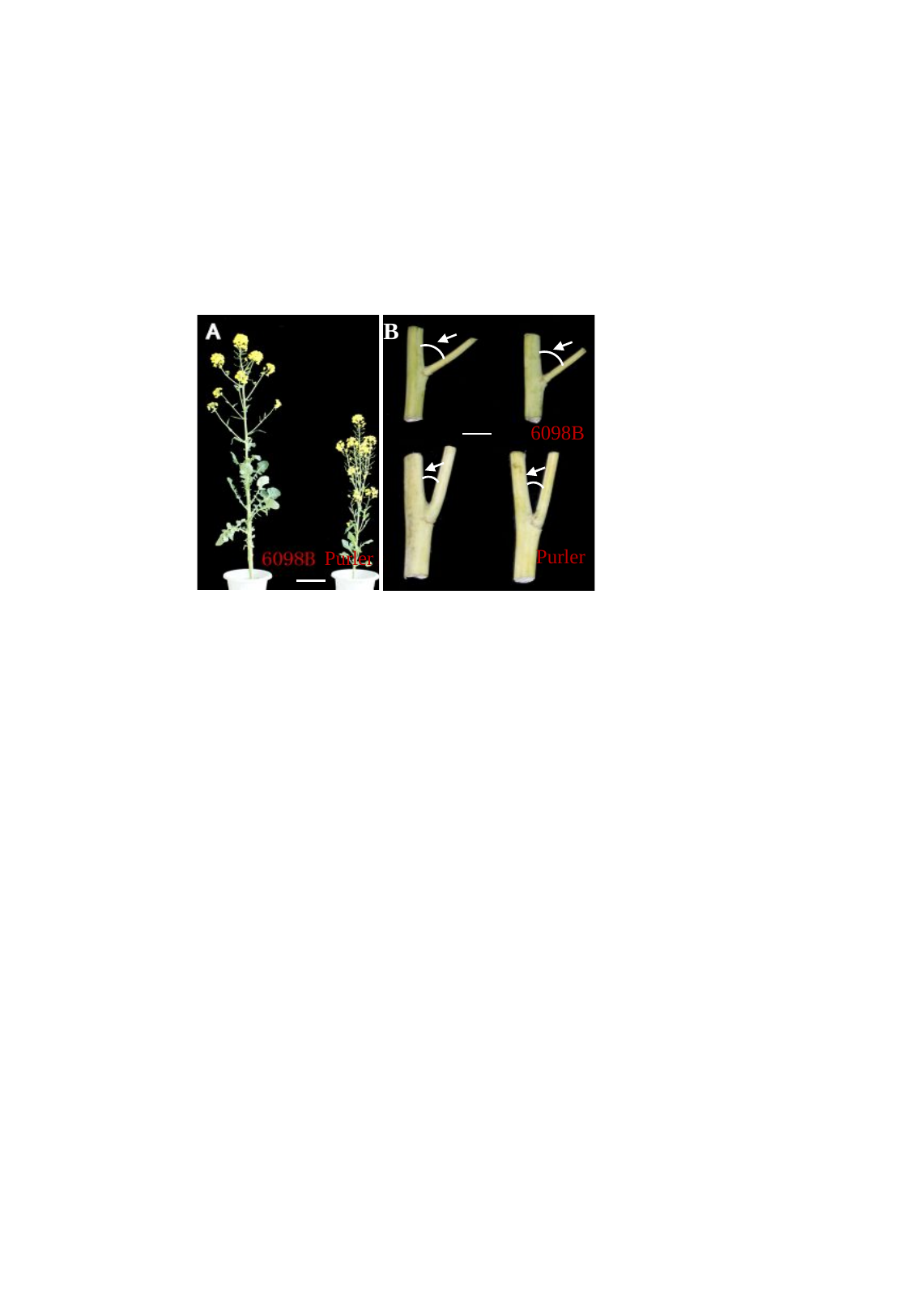

B
6098B
Purler
Purler

Supplement: Additional file 4: Figure S1. — Phenotypes of two lines with different branch angle. (A) 6098B and Purler lines grown at the middle flowering stage. Bar = 25 cm. (B) The branch angle of 6098B is larger than that of Purler. The arrows indicate the different branch angle of two lines. Bar = 2 cm. (PPTX 145 kb) [file 12870_2016_852_MOESM4_ESM.pptx]
